# Hepatitis C reinfection in patients with sustained virologic response at a national hospital in Peru, 2024

**DOI:** 10.17843/rpmesp.2025.422.14428

**Published:** 2025-06-09

**Authors:** Rommel Zambrano-Huailla, Omar Rodríguez Lozano, César Castro Villalobos, Jorge Garavito-Rentería, César Cabezas

**Affiliations:** 1 Liver Unit, Gastroenterology Department, Arzobispo Loayza National Hospital, Lima, Peru Liver Unit Gastroenterology Department Arzobispo Loayza National Hospital Lima Peru; 2 National Institute of Health, Lima, Peru. National Institute of Health Lima Peru

To the Editor. Despite advances in the availability of direct-acting antivirals (DAAs) and in achieving a satisfactory sustained virologic response (SVR), hepatitis C virus (HCV) continues to pose a significant challenge to global public health due to its persistent burden of morbidity and mortality ^1^. In this context, micro-elimination strategies targeting vulnerable populations, such as people on hemodialysis, those coinfected with HIV/HCV, people who inject drugs (PWID), prisoners, indigenous communities, and migrants from countries with high prevalence, have proven effective and have contributed significantly to progress toward the elimination goals set by the World Health Organization (WHO) for 2030, which include an 80% reduction in new HCV cases and a 65% reduction in HCV-related deaths ^2^.

Although rates of HCV reinfection after successful treatment are rare, they remain high among individuals with ongoing risk behaviors such as PWID and men who have sex with men (MSM) ^3^. For this reason, current international guidelines suggest clinical follow-up after HCV cure with a quantitative HCV-RNA test at least every six months in high-risk populations ^3^^,^^4^. Reinfections, particularly in high-risk populations, compromise the HCV elimination goals set by the WHO for 2030. It is important to note that reports on reinfection rates after DAA therapy in Latin America are scarce.

We conducted a study to determine the rates of HCV reinfection in patients with SVR at the Arzobispo Loayza National Hospital in Lima, Peru, in 2024. Reinfection was assessed using quantitative HCV-RNA (hepatitis C virus RNA) tests and clinical follow-up. For an accurate estimate of the incidence density of HCV reinfection, we used cases considered to be reinfected during cumulative follow-up in a cohort of patients. The total time at risk for reinfection ranged from the confirmatory SVR test or the end of DAA treatment.

**Figure 1:**
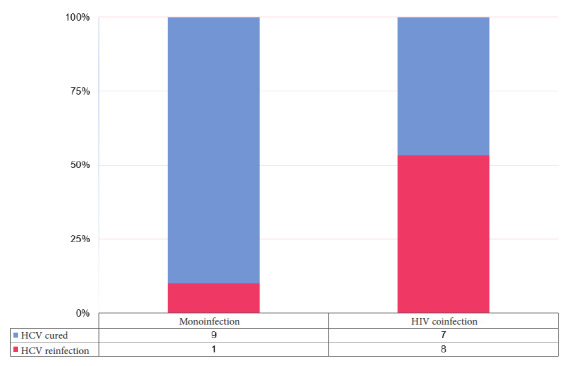
Proportion of patients with hepatitis C virus reinfection according to HIV infection.

We evaluated 25 of 68 patients with SVR. Due to insufficient confirmatory evidence in clinical practice, individuals with greater vulnerability were prioritized. The average age was 48 years (± 16 years), 76% (n=19) were male, and 24% (n=6) were female. The average follow-up of all participants was 27 months (± 18 months); 15 patients (60%) had HIV coinfection and 87% had an undetectable viral load. Nine cases of HCV reinfection were identified, with a reinfection incidence of 59.3 per 100 person-years (95% CI: 21.3-83.9), with a median time to HCV reinfection from SVR of 22 months (range 10-27 months). The incidence of reinfection was higher among people with HIV coinfection (65.4 per 100 person-years, 95% CI: 23.6-88.3) compared with people without HIV (10 per 100 person-years, 95% CI: 0.5-37.4) (Figure 1). With regard to risk factors, all patients with HCV reinfection were MSM, and 60% engaged in high-risk sexual behaviors (multiple sexual partners and group sex).

These preliminary results suggest a higher-than-expected reinfection rate, highlighting the need for additional strategies to control HCV transmission in vulnerable populations. In Latin America, where information is limited, Hernán Vergara-Samur *et al*. ^5^^)^ described a low reinfection rate in Colombia (6.3%). A recent study by Jaime A. Collins *et al*. ^6^^)^ reports a lower percentage of HCV reinfection in HIV patients (11%) treated with DAA. Although PWID is a known risk factor for reinfection, there were no PWID in our sample. This shows a different behavior in HCV transmission that should be explored in future studies. It is important to note that antibodies against hepatitis C do not protect against reinfection.

In conclusion, addressing treatment and follow-up care for HCV cure can improve outcomes toward achieving elimination goals by 2030. The lack of clear guidelines for follow-up care for HCV-cured patients in Peru underscores the need for updated public health policies. We believe that new recommendations on surveillance of populations vulnerable to reinfection could play an important role in developing strategies to control transmission.
