# Supplementary material for: Hepatitis C reinfection in patients with sustained virologic response at a national hospital in Peru, 2024
Source: Rev Peru Med Exp Salud Publica. 2025 Jun 9;42(2):223–4. doi: 10.17843/rpmesp.2025.422.14428 (PMC12377881; doi:10.17843/rpmesp.2025.422.14428)
Supplement: Translation

# Translation: Reinfección por hepatitis C en pacientes con respuesta virológica sostenida en un hospital nacional del Perú, 2024

**Authors:** Rommel Zambrano-Huailla, Omar Rodríguez Lozano, César Castro Villalobos, Jorge Garavito-Rentería, César Cabezas

**Affiliations:** 1 Unidad de Hígado, Servicio de Gastroenterología, Hospital Nacional Arzobispo Loayza, Lima, Perú; 2 Instituto Nacional de Salud, Lima, Perú.

Sr. Editor. A pesar de los avances en la disponibilidad de antivirales de acción directa (AAD) y en la obtención de una respuesta virológica sostenida (RVS) satisfactoria, el virus de la hepatitis C (VHC) continúa representando un desafío importante para la salud pública a nivel mundial, debido a su persistente carga de morbilidad y mortalidad ^1^. En este contexto, las estrategias de microeliminación dirigidas a poblaciones vulnerables, como personas en hemodiálisis, coinfectadas con VIH/VHC, usuarios de drogas inyectables (PWID), personas privadas de libertad, comunidades indígenas y migrantes provenientes de países con alta prevalencia, han demostrado ser efectivas y han contribuido significativamente al avance hacia las metas de eliminación establecidas por la Organización Mundial de la Salud (OMS) para 2030, que incluyen una reducción del 80 % en los nuevos casos de VHC y del 65 % en las muertes relacionadas con esta infección ^2^.

Aunque las tasas de reinfección por VHC después de un tratamiento exitoso son raras, siguen siendo elevadas entre los individuos con conductas de riesgo continuo como los PWID y hombres que tienen sexo con hombres (HSH) ^3^. Por esta razón, las guías internacionales actuales sugieren el seguimiento clínico después de la cura del VHC con una prueba cuantitativa de HCV-RNA al menos cada seis meses en poblaciones de alto riesgo ^3^^,^^4^. La reinfección, particularmente en poblaciones de alto riesgo, compromete las metas de eliminación del VHC establecidas por la OMS para el 2030. Es importante resaltar que, informes sobre las tasas de reinfección después de la terapia con AAD en América Latina son escasos.

Realizamos un estudio con el objetivo de determinar los porcentajes de reinfección por VHC en pacientes con RVS en el Hospital Nacional Arzobispo Loayza en Lima, Perú en el 2024. La reinfección fue evaluada mediante pruebas cuantitativas de HCV-RNA (ARN del virus de la hepatitis C) además de seguimiento clínico. Para una estimación adecuada de la densidad de incidencia de reinfección por VHC se basó en los casos considerados como reinfección durante el seguimiento acumulado en una cohorte de pacientes. El tiempo total de riesgo de reinfección comprendió desde la prueba confirmatoria de RVS o el final del tratamiento con AAD.

Se evaluaron 25 de 68 pacientes con RVS. Debido a no contar con suficiente prueba confirmatoria en la práctica clínica, se priorizaron a los individuos con mayor vulnerabilidad. El promedio de edad fue de 48 años (± 16 años), el 76% (n=19) fueron varones y el 24% (n=6) mujeres. El seguimiento promedio de todos los participantes fue de 27 meses (± 18 meses); 15 pacientes (60%) presentaron coinfección VIH y el 87% una carga viral indetectable. Se identificó nueve casos de reinfección por VHC, con una incidencia de reinfección de 59,3 por 100 personas-año (IC 95%: 21,3-83,9), con un tiempo mediano hasta la reinfección por VHC desde la RVS de 22 meses (rango de 10-27 meses). La incidencia de reinfección fue mayor entre las personas con coinfección por VIH (65,4 por 100 personas-año, IC 95%: 23,6-88,3) en comparación con las personas sin VIH (10 por 100 personas-año, IC 95 %: 0,5-37,4) (Figura 1). Con respecto a los factores de riesgo, todos los pacientes con reinfección por VHC eran HSH y el 60% tenía conductas sexuales de alto riesgo (múltiples parejas sexuales y sexo grupal).

Estos resultados preliminares sugieren una densidad de reinfección más alta de lo esperado, lo que resalta la necesidad de estrategias adicionales para controlar la transmisión del VHC en poblaciones vulnerables. En Latinoamérica donde la información es limitada, Hernán Vergara-Samur *et al.*^5^ describieron una baja tasa de reinfección en Colombia (6,3%). Un estudio reciente realizado por Jaime A. Collins *et al*^6^ reporta un porcentaje menor de reinfección de VHC en pacientes con VIH (11%) tratados con AAD. A pesar de que PWID es un factor de riesgo conocido para la reinfección, no hubo PWID en nuestra muestra. Esto indica un comportamiento diferente en la transmisión del VHC que debe ser explorado en futuros estudios. Es importante resaltar que los anticuerpos contra la hepatitis C no son protectores contra la reinfección.

**Figura 1:** Proporción de pacientes con reinfección de virus de la hepatitis C de acuerdo a la infección por VIH.

En conclusión, abordar el tratamiento y el seguimiento de la cura del VHC puede mejorar los resultados para alcanzar los objetivos de su eliminación para 2030. La falta de guías claras para el seguimiento de pacientes curados de VHC en Perú subraya la necesidad de políticas de salud pública actualizadas. Creemos que nuevas recomendaciones sobre la vigilancia de poblaciones vulnerables a la reinfección podrían desempeñar un papel importante en el desarrollo de estrategias para controlar la transmisión.
